# Intensive Care Unit–Related Cholangiopathy-Induced Biliary Cast Syndrome Without Liver Transplantation: A Rare Entity

**DOI:** 10.14309/crj.0000000000001269

**Published:** 2024-02-17

**Authors:** Fnu Vikash, Sindhu Vikash, Sammy Ho, Donald Kotler, Sunny Patel

**Affiliations:** 1 Department of Medicine, Jacobi Medical Center, Albert Einstein College of Medicine, Bronx, NY; 2 Division of Gastroenterology, Montefiore Medical Center, Albert Einstein College of Medicine, Bronx, NY; 3 Division of Gastroenterology, Jacobi Medical Center, Albert Einstein College of Medicine, Bronx, NY

**Keywords:** biliary cast syndrome, liver transplantation, cholangiopathy

## Abstract

The development of biliary cast syndrome (BCS) is very rare, mostly documented in patients with liver transplantation. The etiology of BCS is unknown; however, risk factors include post–liver transplant bile duct injury, ischemia, infection, fasting, parenteral feeding, and increased bile viscosity and gallbladder dysmotility. We present the case of a 41-year-old man who developed BCS secondary to a prolonged intensive care unit course without a liver transplant. This case highlights the importance of monitoring patients with protracted intensive care unit course and abnormal aminotransferases to recognize and timely manage cholangiopathy and BCS-related complications.

## BACKGROUND

Biliary cast syndrome (BCS), a rare condition identified in 1975, involves the formation of hardened, dark material resembling a biliary tree. Without intervention, it results in obstruction, cholangitis, and complications such as multiple strictures, ductal dilation, and liver microabscesses.^1^ The incidence among patients undergoing orthotopic liver transplantation ranges from 5% to 18%.^2^ Although limited cases of BCS have been reported in patients without liver transplantation, the exact pathogenesis of biliary cast formation remains unclear.^3,4^ It is likely to be multifactorial, with biliary sludge considered a prerequisite.^5^ We report a rare intensive care unit (ICU)-related cholangiopathy-induced BCS without liver transplantation.

## CASE REPORT

A 41-year-old man with a history of hypertension was admitted to ICU because of intracranial hemorrhage secondary to a hypertensive emergency. The clinical course was complicated by seizures, which was managed with antiepileptics and intubation. The patient developed septic shock secondary to ventilator-associated pneumonia and was started on vasopressors. At the time of extubation, the patient had cardiac arrest preceded by severe agitation and persistent tachycardia. Return of spontaneous circulation was achieved within 13 minutes, and the patient was reintubated.

Throughout the ICU stay, multiple sedatives, including dexmedetomidine, fentanyl, ketamine, midazolam, and propofol drips, along with adjuvant sedatives such as diazepam, methadone, gabapentin, phenobarbital, quetiapine, valproate, melatonin, and lorazepam, were used to manage breakthrough episodes of severe agitation. Despite 6 months of ICU care, the patient failed multiple spontaneous breathing trials, leading to the placement of a tracheostomy and a percutaneous endoscopic gastrostomy tube, followed by transfer to the general floors.

**Figure 1.**
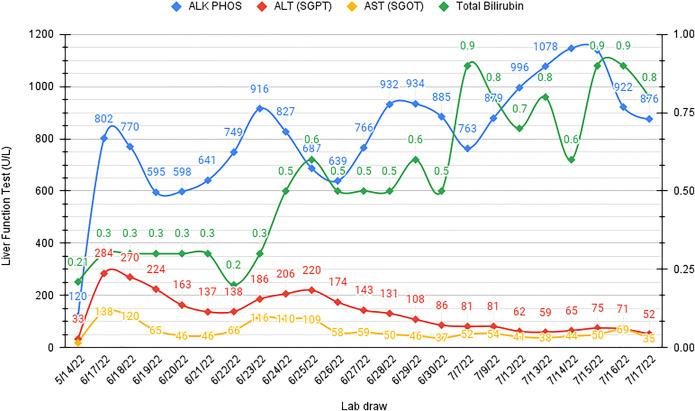
The cholestatic pattern of LFTs with increasing trends of ALK PHOS (reference range: 44–147 IU/L). However, post-ERCP ALT (reference range: 0–40 IU/L) and AST (reference range: 0–40 IU/L) started improving, and total bilirubin remained normal (reference range: 0.1–1.2 mg/dL). ALK PHOS, alkaline phosphatase; ALT (SGPT), alkaline phosphatase (serum glutamic pyruvic transaminase); AST (SGOT), aspartate aminotransferase (serum glutamic oxaloacetic transaminase); ERCP, endoscopic retrograde cholangiopancreatography; LFTs, liver function tests.

**Figure 2.**
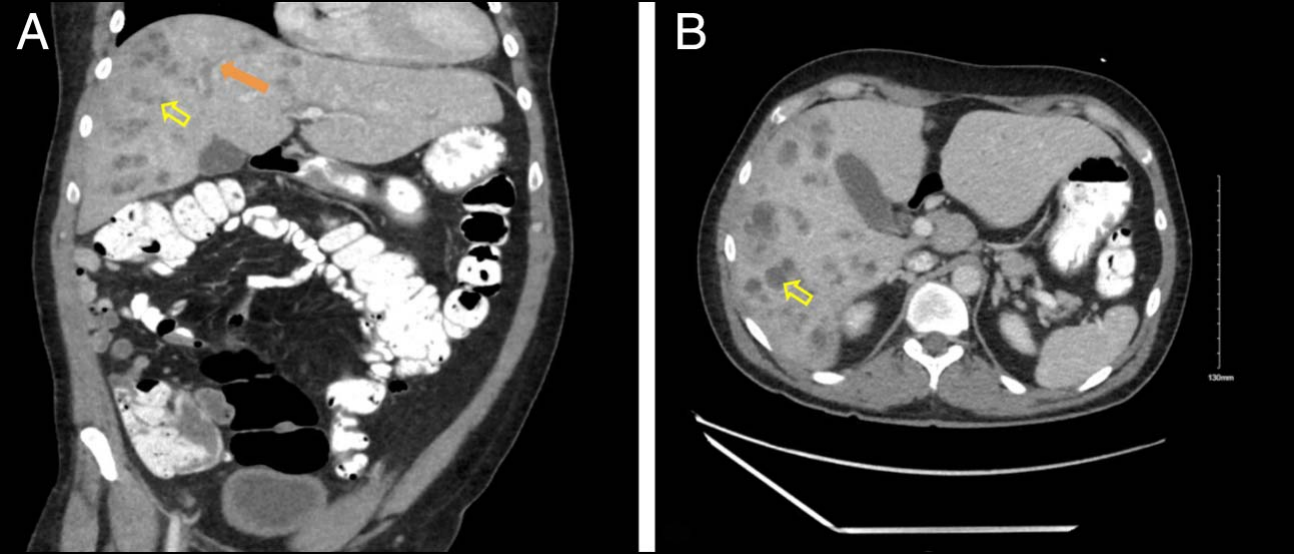
Computed tomography of the abdomen with contrast coronal section (A) and axial section (B) shows multiple ill-defined right hepatic hypodensities (yellow open arrows) and intrahepatic duct dilation (orange arrow).

Two days after being on floor, the patient complained of abdominal pain and had a fever of 100.4°F. Liver function tests (LFTs) revealed a cholestatic pattern (Figure 1). Contrast-enhanced computed tomography of the abdomen indicated multiple ill-defined hypodensities predominantly in the right lobe, consistent with a hepatic abscess and intrahepatic duct dilation (Figure 2). Biopsy results confirmed a *Klebsiella aerogenes*–positive liver abscess with abundant neutrophils, necrotic debris, and bile, confirming its biliary origin.

**Figure 3.**
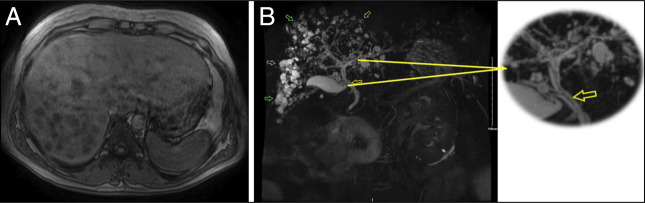
Magnetic resonance imaging with magnetic resonance cholangiopancreatography of the abdomen axial section (A) and coronal section (B) shows innumerable hyperintense and hypointense lesions with peripheral enhancement (green open arrow) and linear T2-hypointense structure in the RHD, CHD, CBD (yellow open arrow). CBD, common bile duct; CHD, common hepatic duct; RHD, right hepatic duct.

Further investigations, including magnetic resonance cholangiopancreatography, revealed innumerable T2-hyperintense/T1-hypointense lesions and a T2-hypointense linear structure in the right hepatic duct, common hepatic duct (CHD), and common bile duct (CBD) (Figure 3). Endoscopic ultrasound demonstrated a mildly dilated CHD (7 mm) with an isoechoic linear structure and scattered areas of hyperechoic filling defect, alongside a normal-sized CBD (5 mm) (Figure 3).

**Figure 4.**
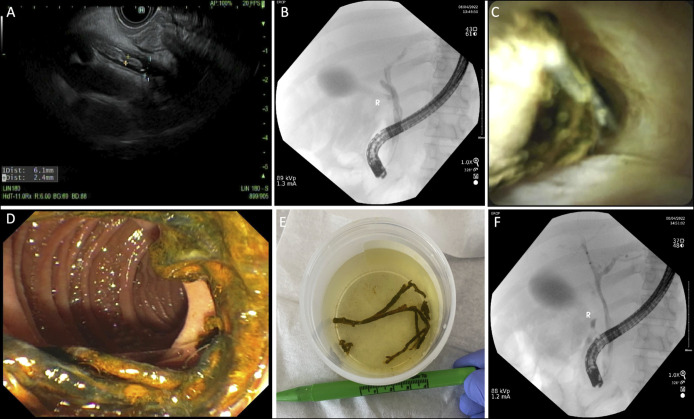
Endoscopic ultrasound revealed the hyperechoic tubular structure in the common bile duct (A). A cholangiogram demonstrates a filling defect in CBD, CHD, and RHD (B). Cholangioscopy confirmed an obstructing linear object in the RHD (C), which was successfully retrieved (D). Gross examination shows a tan-brown bifurcated linear specimen measuring 1 × 10.5 cm, consistent with the biliary cast (E). Subsequent occlusion cholangiogram confirmed excellent contrast drainage and duct clearance (F). CBD, common bile duct; CHD, common hepatic duct; RHD, right hepatic duct.

Subsequent endoscopic retrograde cholangiopancreatography (ERCP) with cholangiogram confirmed a filling defect in right hepatic duct, CHD, and CBD (Figure 3). Single-user SpyGlass Cholangioscopy directly visualized the bile duct, confirming an obstructing linear object (Figure 4). A balloon sweep removed a 10- to 12-cm long tan-brown bifurcated structure with draining purulent material, along with multiple irregular soft-tissue strands submitted in 3 cassettes embedded in formalin (Figure 4). After cast removal, an occlusion cholangiogram confirmed a cleared biliary duct and excellent contrast drainage (Figure 4).

**Figure 5.**
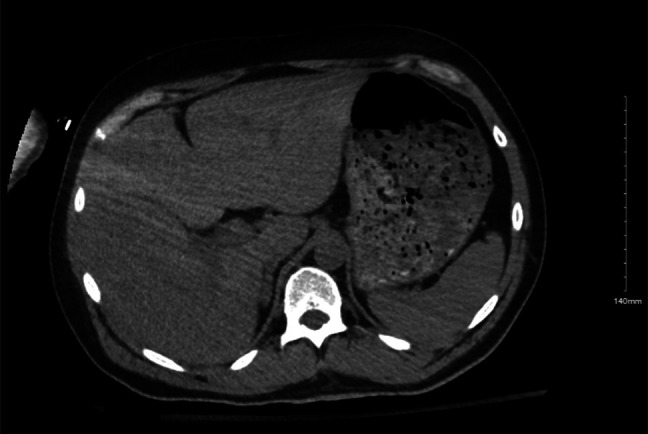
Computed tomography of the abdomen and pelvis (axial section) shows unenhanced liver without biliary duct dilation.

Histopathology of the specimen demonstrated a biliary cast with focal pigmented debris, acute purulent inflammation, and minute strips of ductal epithelium with focal rare reactive atypia. Post-ERCP improvement in LFTs and symptoms was noted (Figure 1). Three weeks later, a post-ERCP computed tomography of the abdomen demonstrated an unenhanced liver without biliary duct dilation (Figure 5).

## DISCUSSION

This case report underscores an uncommon scenario of a prolonged 6-month ICU course, marked by septic shock, cardiac arrest, administration of multiple sedatives, and sustained total parenteral nutrition. Despite the absence of pre-existing liver disease or evidence of ischemic liver injury, a distinctive complication emerged. Literature on the biliary cast is limited, primarily focusing on its post-transplant pathology. Limited case reports discuss secondary sclerosing cholangitis in critically ill patients with underlying BCS.^6^ To date, only 1 case report has been published, which outlines a patient developing a liver abscess with underlying BCS after liver transplantation.^7^ We present a unique case of liver abscess in a patient diagnosed with underlying BCS without liver transplantation.

The pathophysiology of BCS remains unclear. Contributing factors in non–liver transplant patients may include hepatic infarction, fasting-related gallbladder hypocontractility, biliary infection, and biliary ischemia because of sepsis-induced hypotension.^8–10^ ICU patients tend to develop gallbladder sludge contributing to BCS.^11^ Prolonged fasting in an ICU exacerbates gallbladder hypocontractility, fostering the formation of sludge.^12^ Although our patient was exposed to similar risk factors, no biliary sludge was detected on imaging. Another probable mechanism contributing to BCS is lower hepatosplanchnic flow and oxygen uptake because of vasopressor use and cardiac arrest. Furthermore, mechanical ventilation with positive end-expiratory pressures exceeding 10 cm H_2_O contributes to microcirculatory ischemia within the hepatosplanchnic vascular plexus, leading to direct ischemic injury to the biliary tree and cast formation.^13,14^ Drug-induced injury, such as ketamine-induced cholestatic liver injury, may also play a role, as demonstrated by a single-center study in Zurich.^15^ Another potential contributing factor is prolonged sedation, which may lead to the dilation of the bile duct and an increase in biliary pressure.^16,17^ Therefore, we propose that biliary dyskinesia secondary to prolonged sedation is also a predisposing factor for BCS in our case. These factors lead to cholangiocyte necrosis, cast formation, recurrent infection, and abscess formation. If untreated, persistent inflammation and scarring culminate in additional biliary obstruction and cirrhosis.

No established management guideline exists for patients with BCS. Literature indicates that biliary casts pose challenges for endoscopic therapy, often resulting in unsuccessful outcomes.^9,18^ Nevertheless, recent technological advancements, improved techniques, and interventional skills have increased endoscopic success rates, particularly in post-OLT patients with cast formation by 25%.^19^ We advocate prioritizing ERCP over laparotomy, supported by our own experience. In challenging cases, nasobiliary drainage is a potential option, although data are limited.^20^

In conclusion, the etiology of BCS is multifactorial, and the precise pathogenesis is poorly understood. Close monitoring of LFTs in patients with prolonged ICU stays is crucial for the early identification of cholangiopathy and BCS-related complications. The development of a precise management algorithm for this condition is still underway, highlighting the need for further study and exploration of this entity.

## DISCLOSURES

Author contributions: F. Vikash: conceptualization of idea, direct patient care, chart revision, writing manuscript draft, and revisions. S. Vikash: chart revision, writing manuscript draft, and revisions. S. Ho and D. Kotler: manuscript critical review. S. Patel: manuscript critical review, interventionist, and is the article guarantor.

Financial disclosure: None to report.

Informed consent was obtained for this case report.
